# Draft Genome Sequence of *Clostridium aceticum* DSM 1496, a Potential Butanol Producer through Syngas Fermentation

**DOI:** 10.1128/genomeA.00258-15

**Published:** 2015-04-30

**Authors:** Yoseb Song, Soonkyu Hwang, Byung-Kwan Cho

**Affiliations:** a Department of Biological Sciences and KAIST Institute for the BioCentury, Korea Advanced Institute of Science and Technology, Daejeon, Republic of Korea; b Intelligent Synthetic Biology Center, Daejeon, Republic of Korea

## Abstract

*Clostridium aceticum* DSM 1496 is a Gram-negative anaerobic chemolithoautotrophic acetogenic bacterium that is capable of producing commodity chemicals from syngas fermentation. In this study, we report the draft genome sequence of the *C. aceticum* DSM 1496 strain (4.16 Mb) to elucidate the syngas fermentation metabolic pathway.

## GENOME ANNOUNCEMENT

Syngas-utilizing acetogenic bacteria have attracted a lot of attention because of their ability to produce value-added chemicals with higher catalyst specificity, lower energy cost, and greater resistance to catalyst poisoning than traditional thermochemical processing methods, such as Fischer-Tropsch synthesis (1–4). One of the syngas-utilizing acetogens, *Clostridium aceticum* DSM 1496, is the first acetogenic bacterium isolated from sewage sludge proven to grow chemolithoautotrophically by converting carbon dioxide (CO_2_) or carbon monoxide (CO) as key precursors into acetate as the main end product under strict anaerobic conditions (5, 6). Additionally, *C. aceticum* metabolizes various carbon substrates, such as fructose, ribose, glutamate, fumarate, malate, pyruvate, formate, and ethanol (6). Despite this metabolic capability, a lack of genetic information on *C. aceticum* hindered strain engineering to produce the commodity chemicals. In this study, the draft genome sequence of *C. aceticum* DSM 1496 was obtained to study its physiological and metabolic properties.

*C. aceticum* was cultivated under anaerobic conditions in DSMZ medium 135 supplemented with 10% fructose. The isolation and fragmentation of genomic DNA were carried out using the PowerSoil DNA isolation kit (Mo Bio Laboratories, Carlsbad, CA) and Covaris S220 (Covaris, Inc., Woburn, MA), respectively. The fragmented genomic DNA was used to construct an Illumina paired-end library using a TruSeq kit (Illumina, Inc., San Diego, CA), then sequenced using the MiSeq v.2 platform with a 2 × 150-cycle paired-end read recipe. The obtained reads were trimmed on the CLC Genomics Workbench (CLC bio, Aarhus, Denmark) using default parameters, which resulted in a total of 17,994,419 reads with an average read length of 150.22 bp. The retrieved reads were then assembled using the CLC Genomics Workbench (minimum contig length, 606; automatic bubble size, yes; word size, 61; perform scaffolding, yes). The Rapid Annotation using Subsystem Technology server was used to annotate the draft sequence of the strain (7). rRNA and tRNA genes were predicted using RNAmmer 1.2 (8) and tRNAscan-SE 1.31 (9), respectively.

The draft genome sequence of *C. aceticum* DSM 1496 comprises 51 contigs, with 4,158,708 bases, 35.18% G+C content, 4,010 predicted open reading frames, and 44 tRNA genes. Three rRNA genes were also obtained, which are identical to the previously sequenced 16S rRNA genes (10). *C. aceticum* utilizes a six-carbon sugar, such as fructose, to conserve energy compounds and obtain various organic chemicals (6). The draft genome elucidates a glycolysis/gluconeogenesis pathway, pentose phosphate pathway, and glutamate pathway to metabolize various carbon sources (11). Additionally, 11 genes for the reductive acetyl-CoA pathway and 4 genes responsible for the conversion of acetyl-CoA to acetate to obtain ATP were identified, respectively, confirming the ability of the strain to convert CO_2_ or CO to acetate and a complex carbohydrate (12, 13). Interestingly, genes responsible for producing butanol from acetyl-CoA were detected, which has not been reported yet, indicating the potential capability of the strain to produce butanol. With the unraveled genetic contents, the *C. aceticum* DSM 1496 draft genome sequence will help us understand the syngas fermentation metabolic capability of the strain.

### Nucleotide sequence accession numbers. 

The draft genome sequence of *C. aceticum* DSM 1496 has been deposited in the DDBJ/EMBL/GenBank database under the accession no. JYHU00000000. The version described in this paper is the first version, JYHU00000000.1.
